# Morgan County Medical Society

**Published:** 1868-06

**Authors:** C. T. Wilbur

**Affiliations:** Secretary


Society adjourned.

### MORGAN COUNTY MEDICAL SOCIETY.

Society met at 2½ o'clock P.M., in Music Hall, Jacksonville, on Thursday, Feb. 13, 1868. Dr. Henry Jones in the chair.

Minutes of the last meeting were read and approved.

Dr. W. H. H. King reported a case of poisoning from laudanum: On Tuesday night, at 2 o'clock A.M., Dr. Prince and myself were summoned in great haste to see a young lady who, the messenger said, had been taking laudanum, and could not be awakened. With little delay the call was answered. On entering the house, the loud respiration of the patient, who lay in a back room, could be distinctly heard. She was found exhibiting the most prominent symptoms of poisoning from opium: the respiration stertorous; pupils contracted and insensible to light; countenance pale and cadaverous; pulse weak, thread-like, scarcely perceptible; the muscles in a complete state of relaxation; and totally insensible to all external impressions. Two empty vials, holding, respectively, two and three ounces, labeled laudanum, were found on a chair near the bedside, containing about one ounce of liquid, which had the distinct odor of laudanum.

It was stated by residents of the house that the young lady had gone to her room about 10 o'clock P.M., carrying with her two teacups, and that before retiring was engaged in writing. The alarm was given by a young man, a boarder in the house, who had gained access to her room through a window.

An emetic of ant. et potass. tartratis was at once administered, and then the stomach pump was brought into requisition, not waiting for the emetic to act. Three or four ounces of liquid, having the peculiar smell of laudanum, were pumped from the stomach, when it was thoroughly washed out with warm water. Dr. Prince then directed two drachms of extract of belladonna to be dissolved in a teacup of warm water, a tablespoonful to be administered every half hour, in accordance with the theory that the general poisonous action of one narcotic producing dilatation of the pupil will counteract the general poisonous action of another producing contraction. After the administration of four doses of this preparation, the pupils became widely dilated. There being no improvement in the condition of the patient, this remedy was suspended.

Artificial respiration was kept up continuously from 2 o'clock

A.M. until 3 o'clock P.M., it being hoped that if the functions of the lungs and heart could be sustained until the brain had recovered from the influence of the narcotic, that the patient would survive. At 3 o'clock P.M. the patient ceased to breathe, having manifested no symptoms of returning consciousness, except the flexion of the upper and lower extremities and the opening of her eyes twice.

Dr. King closed his remarks by asking the question: "Was the extreme dilatation of the pupils in this case due to the opium or to the belladonna?"

Dr. Prince exhibited and explained the obstetrical bandage of Dr. Hamilton, of Jerseyville.

Dr. Henry Jones related a case of placenta prævia, which had recently occurred in his practice. As an evidence of the rarity of the occurrence among practising physicians, no one of the sixteen physicians present had had in his practice a case of this presentation, except the case related by Dr. Jones.

Dr. Prince remarked that he believed that in this presentation the profession would eventually adopt the maxim, "The most speedy possible delivery."

Dr. Johnson, of Lynnville, reported a singular case of acute nervous disease, about which he had experienced considerable difficulty in a diagnosis. After describing it and relating his treatment, he asked for opinions as to its nature. No opinions were ventured or offered by any of the gentlemen present.

Dr. J. W. Craig read a paper on pneumonia.

Dr. A. H. Kellogg and Dr. E. H. Knight were appointed for essays for the next meeting.

Society adjourned at 4½ o'clock P.M., to meet on the second Thursday in April. C. T. WILBUR, M.D., *Sec'y.*

---

The Morgan County Medical Society met at Music Hall, in Jacksonville, at 2½ o'clock P.M., on Thursday, March 12, 1868, with the President, Dr. Henry Jones, in the chair.

The proceedings of the last meeting were read and approved. Dr. Charles Dutton being about to leave the State, gave notice of his withdrawal from the Society.

Dr. Kellogg, of Jacksonville, read a paper on some of the pathological conditions of typhoid fever. Dr. Knight was not present. Dr. Kellogg was requested to furnish his paper for publication in some medical journal.

Dr. Jones asked, "Did you observe, in autopsies of cases of chronic diarrhœa, any lesions of Peyer's glands?"

Dr. Kellogg replied, "We made some two hundred *post mortems.* A controversy directed my attention to that point especially. Do not remember to have seen typhoid fever without enlargement of the eliptical plates or ulceration, but in chronic diarrhœa I do not remember to have seen any. In one case, there was perforation of the intestine, but never lesion of Peyer's glands. Found lesions of Peyer's glands invariably in typhoid fever."

Dr. Prince asked, "In observation in the cases of malarial fevers, did you find lesions of the intestines?"

Dr. Kellogg remarked, "that he did not remember to have observed any such lesions. Some noted physicians have claimed that the same lesions are found in scarlet fever. I do not pretend to say that they always exist in typhoid fever; but I have always found them."

Dr. Edgar objected to the ordinarily received doctrine that ulceration of Peyer's glands is characteristic of the disease; claiming that the same lesion is found in chronic diarrhœa.

In the discussion and remarks on the treatment of typhoid fever, Dr. Bibb stated that, in certain stages of the disease, he found nitric acid and strychnine very beneficial.

Dr. Jones thought milk was an excellent and safe diet, contrary to the common belief. Typhoid fever seldom proves fatal in children under twelve years of age.

Dr. Fisher believes in supporting the patient. Prescribes milk, to be taken often and with regularity, as a diet. Also uses, as remedies, the turpentine emulsion and diluted nitric acid. A large proportion of patients, if supported with milk diet liberally, will get well without special remedies.

Dr. Henry Jones remarked, "I have seen a good many cases of typhoid fever among children; under 10 years of age it is very rare; between 10 and 30 the most common; after 30 very rare.

Dr. Bibb remarked that, where the dry, fissured tongue is observed, glycerine is an excellent local application.

Dr. Jones suggested the application or administration of tartar emetic in inflammation of the mammæ of females after parturition, in doses of $\frac{1}{16}$ of a grain every hour from the first appearance of the disease.

Dr. Fisher remarked, that he had used and could confirm Dr. Jones' use of this remedy.

Drs. Lucas and McVey were appointed for essays for the next meeting.

At 5 o'clock P.M., meeting adjourned, to meet the second Thursday in April.

C. T. WILBUR, M.D., *Secretary.*

